# Early On-Treatment Prediction of the Mechanisms of Acquired Resistance to EGFR Tyrosine Kinase Inhibitors

**DOI:** 10.3390/cancers14061512

**Published:** 2022-03-15

**Authors:** Yu-ra Choi, Youngnam Cho, Seog-Yun Park, Sunshin Kim, Myungsun Shin, Yongdoo Choi, Dong Hoon Shin, Ji-Youn Han, Youngjoo Lee

**Affiliations:** 1 Division of Translational Science, Research Institute, National Cancer Center, Goyang 10408, Korea; 74217@ncc.re.kr (Y.-r.C.); yncho@ncc.re.kr (Y.C.); dhshin@ncc.re.kr (D.H.S.); 2 Genopsy Inc., Seoul 07573, Korea; 3 Department of Pathology, National Cancer Center, Goyang 10408, Korea; 11740@ncc.re.kr; 4 Division of Precision Medicine, Research Institute, National Cancer Center, Goyang 10408, Korea; ksunshin@ncc.re.kr (S.K.); jymama@ncc.re.kr (J.-Y.H.); 5 Division of Convergence Technology, National Cancer Center, Goyang 10408, Korea; audtns3232@ncc.re.kr (M.S.); ydchoi@ncc.re.kr (Y.C.); 6 Center for Lung Cancer, National Cancer Center, Goyang 10408, Korea; 7 Division of Hematology and Oncology, Department of Internal Medicine, National Cancer Center, Goyang 10408, Korea

**Keywords:** lung CANCER, DRUG resistance, *EGFR* mutation, tyrosine kinase inhibitor, *C-MET* amplification

## Abstract

**Simple Summary:**

*EGFR* T790M-positive clones expand in treated by EGFR inhibitors. *C-MET* amplified clones expand in HCC827 lung cancer cells treated by EGFR inhibitors. Early on-treatment kinetics of the resistance-related gene predict the resistance mechanism.

**Abstract:**

Background: Prediction of resistance mechanisms for epidermal growth factor receptor-tyrosine kinase inhibitors (EGFR-TKIs) remains challenging. Thus, we investigated whether resistant cancer cells that expand shortly after EGFR-TKI treatment would eventually cause the resistant phenotype. Methods: We generated two *EGFR*-mutant lung cancer cell lines resistant to gefitinib (PC9GR and HCC827GR). The parent cell lines were exposed to short-term treatment with gefitinib or paclitaxel and then were assessed for *EGFR* T790M mutation and *C-MET* expression. These experiments were repeated in vivo and in clinically relevant patient-derived cell (PDC) models. For validation in clinical cases, we measured these gene alterations in plasma circulating tumor DNA (ctDNA) before and 8 weeks after starting EGFR-TKIs in four patients with *EGFR*-mutant lung cancer. Results: T790M mutation was only detected in the PC9GR cells, whereas *C-MET* amplification was detected in the HCC827GR cells. The T790M mutation level significantly increased in PC9 cells after short-term treatment with gefitinib but not in the paclitaxel. *C-MET* mRNA expression was only significantly increased in gefitinib-treated HCC827 cells. We confirmed that the *C-MET* copy number in HCC827 cells that survived after short-term gefitinib treatment was significantly higher than that in dead HCC827 cells. These findings were reproduced in the in vivo and PDC models. An early on-treatment increase in the plasma ctDNA level of these gene alterations was correlated with the corresponding resistance mechanism to EGFR-TKIs, a finding that was confirmed in post-treatment tumor tissues. Conclusions: Early on-treatment kinetics in resistance-related gene alterations may predict the final mechanism of EGFR-TKI resistance.

## 1. Introduction

Overexpression of epidermal growth factor receptor (EGFR) has been well known to be implicated in the pathogenesis of lung cancer [1]. EGFR tyrosine kinase inhibitors (TKIs) were the first molecular targeted drugs leading to a paradigm shift in the management of patients with advanced non-small cell lung cancer (NSCLC) [2]. Patients with lung cancer harboring drug-sensitive mutations on the *EGFR* gene, such as exon 19 deletion or exon 21 L858R mutations, show dramatic and durable responses to EGFR-TKIs [3]. However, almost all lung cancer cases that initially respond well to these drugs ultimately develop drug resistance and disease progression. In the last decade, several major mechanisms to drive resistance to EGFR-TKIs have been identified through translational research. The most common resistance mechanism of first- or second-generation EGFR-TKIs is a secondary T790M mutation in exon 20 of *EGFR,* as detected in 50–60% of resistant tumors [4,5,6]. *C-MET* amplification is the second most common mechanism, ranging from 5% to 22% of tumors resistant to EGFR-TKIs [7,8]. Other minor resistance mechanisms include mutations in *PIK3CA MAPK1 HER2,* and *BRAF*, epithelial–mesenchymal transition (EMT), and small cell lung cancer transformation [9].

Although the mechanisms of acquired resistance to EGFR-TKI are well known, it remains a challenge to predict the development of specific resistance mechanisms upon EGFR-TKI treatment. Identifying EGFR-TKI resistance before or shortly after the treatment would be helpful to prevent or delay the resistance that leads to disease progression. Many preclinical and clinical studies suggest that some drug-resistant cancer cell clones already exist before exposure to the anti-cancer drug and survive during the course of treatment [8,10,11,12,13,14,15]. In accordance with previous studies, we also reported that 25% of patients with *EGFR*-mutated lung cancer had the T790M resistance mutation before EGFR-TKI treatment, and this pre-existing T790M mutation may negatively affect the drug’s efficacy [12]. However, it is challenging for a conventional sequencing method to detect these latent resistance mutations that exist at a minor frequency. Additionally, if multiple resistance-associated gene alterations exist simultaneously, knowing which resistance mechanism will be finally selected is challenging. Thus, we hypothesized that among several pre-existing cancer cell clones carrying different resistance mechanisms, some resistant cell clones that expand shortly after EGFR-TKIs would eventually cause the resistant phenotype. In this study, we investigated whether resistance clones significantly increase shortly after exposure to EGFR-TKIs and determine the final phenotype of the resistance to EGFR-TKIs using in vitro, in vivo, and ex vivo *EGFR*-mutant tumor models. We further tested this hypothesis in patients who were diagnosed with advanced *EGFR*-mutated NSCLC and treated with EGFR-TKIs by monitoring early dynamic changes of resistance-associated gene alterations during treatment using plasma circulating tumor DNA (ctDNA). Our study focused on developing two main resistance mechanisms: *EGFR* T790M mutation and *C-MET* amplification.

## 2. Materials and Methods

### 2.1. Cell Line and Reagents

HCC827 and PC9 cell lines were purchased from the Korean Cell Line Bank (Seoul, Korea) and RIKEN BioResource Center cell bank (Ibaraki, Japan), respectively. H4006, A549, and H1975 cell lines were obtained from American Type Culture Collection (Manassas, VA, USA). Cells were maintained in RPMI-1640 supplemented with 10% fetal bovine serum. Gefitinib was purchased from LC Laboratories (Woburn, MA, USA). AZD9291, PHA665752, and Afatinib were purchased from Selleckchem (Houston, TX, USA).

### 2.2. Establishment of Gefitinib-Resistant Cell Lines

Gefitinib-resistant cells were established by continuously exposing parent cells to increasing drug concentrations. Beginning at 0.01 μM, the exposure drug dose was doubled until a final concentration of 1.0 μM. Drug-resistant phenotypes were confirmed using cell viability assays. PC9GR and HCC827GR cells were the resistant cells from PC9 and HCC827 cells, respectively.

### 2.3. Establishment of Patient-Derived Cells (PDCs)

Pleural effusions were obtained from patients (female, 65 years old) and collected in sterile sample cups. Samples were transferred to conical tubes and centrifuged at 120× *g* for 10 min at room temperature (RT). The cell pellet was resuspended in RPMI and carefully layered onto LSM (lymphocyte separation medium; #091692249; MP Biomedicals, Illkirch, France). After centrifugation at 400× *g* for 30 min at RT, the interphase layer between RPMI and LSM including tumor cells was harvested and washed with RPMI. The washed cells were cultured in AR-5 medium (5% fetal bovine serum, 1× GlutaMAX (Thermo Fisher Scientific, Waltham, MA, USA), 1× ITS (insulin–transferrin–selenium, Thermo Fisher Scientific), 1% penicillin/streptomycin, 50 nM hydrocortisone, 1 mM sodium pyruvate and 1 ng/mL EGF in RPMI) at 37 °C in a 5% CO_2_ atmosphere.

### 2.4. Cell Viability Assay

The cells were cultured in gefitinib-free medium for 2 weeks before testing. The cells were then seeded at a density of 4 × 10^3^ cells/well in 96-well plates. After 24 h, the cells were exposed to different concentrations of gefitinib and further incubated for 48 h. The cells were then washed with phosphate-buffered saline, and cell viability was measured using the CellTiter 96^®^ AQueous One Solution Cell Proliferation Assay (Promega, Madison, WI, USA) according to the manufacturer’s instructions.

### 2.5. Direct Sequencing

Genomic DNA was extracted from cells using the phenol-chloroform method. Polymerase chain reaction (PCR) was performed using 1 μL of the extracted genomic DNA, SG-PCR Premix-EX mix (SG-P004EX; SG-Bio), and 10 pmol of the primers in a final volume of 20 μL. The primers are shown in Table S1. The PCR cycling parameters were as follows: 95 °C for 5 min, 35 cycles at 95 °C for 30 s, 60 °C for 30 s, and 72 °C for 30 s, followed by a final step at 72 °C for 10 min. After the PCR products were purified, they were directly sequenced by Cosmogenetech, Inc. (Seoul, Korea) according to the manufacturer’s instructions.

### 2.6. Droplet Digital PCR

Droplet digital PCR (ddPCR) was performed using a QX200 Droplet Digital PCR system (Bio-Rad, Hercules, CA, USA). Reactions were performed in 10 μL of ddPCR 2× Master mix, 1 μL of 20× primer, and TaqMan Probe mix (Bio-Rad) (for the T790M mutation: #1863103; MET CNV FAM: #10031240; HEX: #10031243), 8 μL of nuclease-free water, and 1 μL of extracted genomic DNA in a final volume of 20 μL. Each sample was transferred to the middle wells of the cartridge, and 70 μL of droplet generation oil was added to the lower wells. Next, the cartridge was placed into the droplet generator to generate each sample-containing oil droplet; 40 μL droplets of each sample were transferred to the wells of a 96-well PCR plate. The PCR cycling parameters were as follows: 95 °C for 10 min, 40 cycles at 94 °C for 30 s, 58 °C for 1 min, and 98 °C for 10 min. After PCR was complete, the 96-well PCR plate was loaded into the droplet reader to read TaqMan Probe fluorescence in individual droplets. After that, we used QuantaSoft software to analyze the data based on the results from no template control wells with a threshold. Based on a previous study, a limit of detection (LOD) of the ddPCR assays for T790M mutation was determined as 0.05% [16].

### 2.7. Quantitative Real-Time PCR (RT-PCR)

Total RNA was prepared using TRIzol (Ambion, Austin, TX, USA) according to the manufacturer’s protocol. cDNA was synthesized from total RNA using Superscript II (Invitrogen, Carlsbad, CA, USA) (Table S2). Gene expression was investigated using quantitative real-time PCR (qPCR) on a Lightcycler^®^ 480 instrument (Roche, Basel, Switzerland), and all the reactions were run in triplicate. Reactions were performed in 2× Sensi FAST SYBR No-ROX mix (Bioline, London, UK), 8 μL of nuclease-free water, 1 μg of cDNA, and 10 pmol of the primers in a final volume of 20 μL. All the primers were ordered from Cosmogenetech. Gene expression was normalized to the housekeeping gene glyceraldehyde 3-phosphate dehydrogenase.

### 2.8. Immunoblotting

Cells were washed with cold phosphate-buffered saline (PBS) and harvested with radioimmunoprecipitation assay lysis buffer containing phosphatase inhibitor cocktail set V (#524629; MERCK, Kenilworth, NJ, USA) and protease inhibitor cocktail set III (#535140; MERCK). Whole-cell lysates were separated using sodium dodecyl sulfate-polyacrylamide gel electrophoresis and were blotted onto polyvinylidene fluoride membranes (#10600030; Amersham, Little Chalfont, UK). After blocking with 5% skim milk in Tris-buffered saline buffer (pH 8.0) with 0.1% Tween-20, the membrane was incubated with the primary antibody overnight (Cell Signaling Technology, Danvers, MA, USA; anti-pEGFR: #3777S; anti-EGFR: #4267S; anti-pMET: #3077S; anti-MET: #8198S; anti-pAKT: #9271S; anti-AKT: #9272S; anti-pERK: #4370S;anti-ERK: #4695S; anti-E-CADHERIN: #3195S; anti-VIMENTIN: #5741S). After rinsing with Tris-buffered saline buffer, the membrane was incubated with a secondary antibody for 1 h and washed, followed by visualization using electrochemiluminescence (SuperSignal™ West Femto Maximum Sensitivity Substrate; Thermo Fisher Scientific; #34095) and a LAS-3000 detection system (Fujifilm, Tokyo, Japan).

### 2.9. Colorimetric Detection of ctDNA Mutations

The probe sequences were designed using the UCSC Genome Browser. Biotinylated probes were synthesized by Macrogen (Seoul, Korea) (Table S3). Initially, positively charged polyethyleneimine-conjugated nanowires (PEI-mNWs) (Genopsy Inc., Seoul, Korea; 5 µg/mL) were added to 200 µL fragmented DNA samples and mixed for 30 min at RT to allow DNA-NW complex formation. DNA-NW complexes were used after heating at 95 °C for 1 min. Biotin-labelled probe mix (1 pM) was added to the resulting solution and incubated additionally for 20 min at RT, followed by the addition of 2 μg/mL of horseradish peroxidase (HRP)-labelled streptavidin (st)-conjugated nanoparticles (NPs) (HRP-st NPs) (Genopsy Inc., ) and incubation for 5 min at RT. After precipitation, 25 µL of 10 mM 3,3′,5,5′-tetramethylbenzidine, 25 µL of 0.1 M H_2_O_2_, and 200 µL of 0.2 M sodium acetate trihydrate buffer (pH 5.0) were added to the resulting sample. The optical densities (ODs) of the samples were measured using absorbance at 490–800 nm and an Epoch UV-Vis spectrophotometer (BioTek, Winooski, VT, USA). The signal associated with mutation was determined by subtracting the absorbance at 500 nm from the absorbance at 650 nm (ΔOD 650−500).

### 2.10. Synthesis of Anti-C-MET Antibody-Fluorophore Conjugate

For the analysis of C-MET expression on the surface of the cells, anti-C-MET antibody-fluorophore conjugate was prepared as below. Onartuzumab (Anti-C-MET antibody BSA, azide free) was purchased from Evitria (Schlieren, Switzerland). Alexa Fluor 647-NHS ester was obtained from Thermo Fisher Scientific (Waltham, MA, USA).

Ornatuzumab (0.5 mg, 3.33 nmol) was reacted with AF647-NHS ester (antibody: dye mole ratio = 1:3.2) in phosphate buffered saline solution (PBS, pH 7.4) for 1 h at 25 °C. The reaction was performed under the light-protected condition with gentle shaking. The unreacted AF647-NHS ester was removed using a PD-10 column (GE healthcare, Chicago, IL, USA). The eluted antibody-dye conjugates were concentrated using Amicon Ultra-0.5 mL centrifugal filter (MWCO 50K, Millipore, Burlington, VT, USA) and stored at 4 °C till further analysis. The number of conjugated dyes per antibody was analyzed to be 1.5.

### 2.11. Flow Cytometry Staining and Cell Sorting

To detect apoptosis and C-MET expression at the same time, we used Annexin-V-FITC apoptosis detection kit (BD Biosciences, San Diego, CA, USA) and C-MET-Alexa 647 fluorescent dye described in Section 2.10 (Materials and Methods). HCC827 and PC9 cells are grown in 100 mm plates and treated with 0.1 μM of Gefitinib and 0.01 μM Paclitaxel for 48 h. After drug treatment, cells were collected with trypsin-EDTA, washed with phosphate-buffered saline and then suspended in Annexin-V binding buffer solution. After being suspended in 200 μL binding buffer, cells were double stained with 3 μL Annexin-V-FITC and 1 μL C-MET-Alexa 647 fluorescent dyes. Cells were incubated for 30 min at RT in the dark and analyzed by FACs. We gated cells according to the expressions of fluorescent dyes and sorted the cells as Annexin-V positive (Annexin-V^+^/C-MET^High+low^) and Annexin-V negative (Annexin-V^−^/C-MET^High^). We centrifuged the sorted cells and extracted DNA for ddPCR experiment.

### 2.12. Mouse Xenograft Studies

We received approval of protocol from the National Cancer Center for mouse xenograft studies with accession number NCC-19-482. We used only 6-week-old BALB/c-nu female mice (*n* = 48) (Orientbio, Korea). The mice were randomly divided into three groups according to weight (average 20–22 g). They had an adaptation period of one week before the injection of cancer cells. We provided cozy bedding to reduce stress for mouse adaptation. Two cell lines (HCC827 and PC9) were injected and three drugs (dimethyl sulfoxide (DMSO), paclitaxel, and gefitinib) were used for treatment. We injected 1 × 10^6^ cells within 50% Matrigel and measured the tumor volume using a caliper twice a week. Tumor sampling was performed when the volume reached approximately 300 mm^3^. We resected some tumors to obtain pre-treatment samples (1st operation) before starting drug treatment. After closely observing the mice for two days for operation site healing, the mice were randomized to receive one of three drug treatments. The mice that died after the 1st operation were not included in the subsequent experiment. Gefitinib was dissolved in DMSO and orally administered 5 days per week at a dose of 20 mg/kg. Paclitaxel was dissolved in DMSO and intraperitoneally injected once a week at a dose of 30 mg/kg. The tumor volume measurement time, intraperitoneal injection or oral administration time of the drug occurred between 3:30 pm and 6:30 pm. The test sequence was randomized each time, and each animal was tested at different times each day. After 1 week of treatment, the tumors were resected (2nd operation) and lysed in lysis buffers for RT-PCR and ddPCR. We injected continuous carbon dioxide (CO_2_) flow into the cage to induce euthanasia. We injected CO_2_ flow rapidly to induce painless death of the mice. After confirming the mouse’s breathing had stopped, the injection of CO_2_ was stopped. All the animal studies were conducted under the guidance of the Institutional Animal Care and Use Committee, and all relevant ethical regulations were followed.

### 2.13. Patient and Tumor Samples

We recruited four patients who were diagnosed with metastatic NSCLC with *EGFR-*sensitive mutations and had started EGFR-TKIs as a first-line treatment at the National Cancer Center (Goyang, Korea). These four patients with a median age of 58 years (range, 46–68) consisted of 2 males and 2 females. Blood samples were collected at baseline and every 8 to 12 weeks during EGFR-TKI treatment. Tumor response was evaluated every 8 to 12 weeks. Tissue samples were also collected to evaluate the acquired resistance mechanism after disease progression. To evaluate the *EGFR* T790M mutation levels and *C-MET* expression in plasma ctDNA, the nanowire-based colorimetric assay was performed. PEI-NWs (5 μg/mL) and diluted plasma (150 μL) were combined and mixed for 20 min at RT to form DNA-NW complexes. The colorimetric assay was performed as described above.

### 2.14. Ethics Approval and Consent to Participate

All the patients provided written informed consent. This study was performed with approval from the National Cancer Center Institutional Review Board (approval number NCC2011-0547). The study was conducted in compliance with the principles of the Declaration of Helsinki, the International Council for Harmonisation of Technical Requirements for Pharmaceuticals for Human Use guidelines and local ethical and legal requirements. The protocol and IC document were approved by the independent institutional review boards of all the participating institutions.

### 2.15. Statistical Analysis

Each experiment was performed at least three times, and all data are presented as the mean ±  SEM. Statistical analysis was performed using two-sample *t*-test. *p*-values ≤ 0.05 were considered significant (**, *p* ≤ 0.05; ns, not significant).

## 3. Results

### 3.1. Resistance Mechanisms in Different Cancer Cell Lines

**Figure 1:**
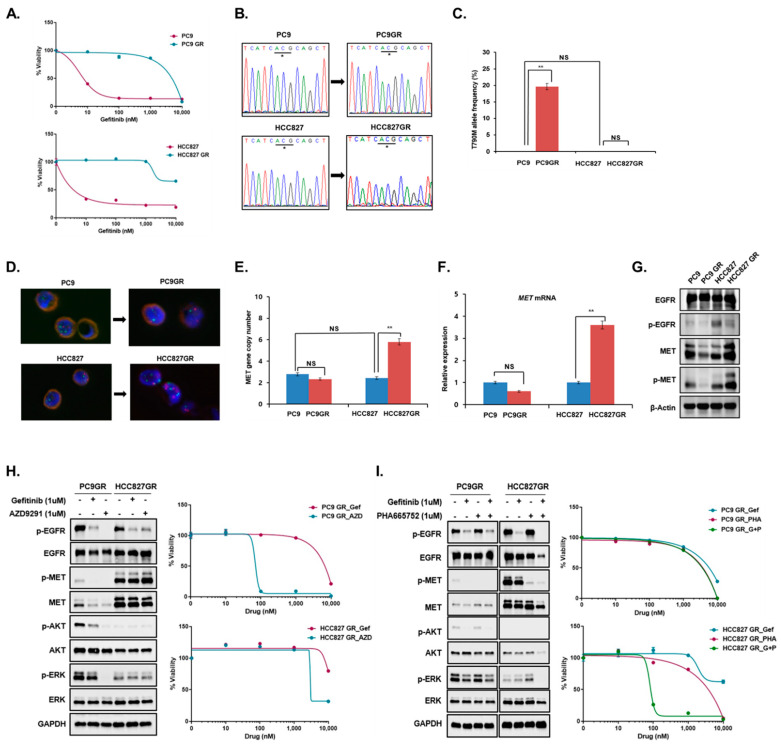
PC9GR and HCC827GR were established, and acquired resistance mechanisms were evaluated. (**A**) Cell viability assays were performed to compare the drug sensitivity of parent cells and EGFR-TKI-resistant cells. (**B**) Direct sequencing and (**C**) ddPCR assays were performed to evaluate the presence of secondary *EGFR* T790M resistance mutations. (**D**) Fluorescence in situ hybridization assays (green signal: centromere 17; red signal: *C-MET* gene) and (**E**) ddPCR assays were performed to evaluate *C-MET* amplification. (**F**,**G**) *C-MET* mRNA expression was measured by RT-PCR, and C-MET protein expression was evaluated by Western blotting. (**H**,**I**) Drug sensitivity to AZD9291 (a selective EGFR inhibitor specific to T790M mutation) and PHA665752 (a selective MET inhibitor) was tested in PC9GR and HCC827GR cells, and then C-MET, EGFR, and EGFR downstream (AKT, ERK) protein expression was evaluated by Western blotting. In all graphs, the error bars represent the mean ± SEM. Statistical analysis was performed using two-sample *t*-test. * codon sequence. ** *p* ≤ 0.05; NS, not significant.

We simultaneously generated two cancer cell lines resistant to EGFR-TKI gefitinib using two lung cancer cell lines with the same *EGFR* mutation (Figure 1A). The molecular mechanism of gefitinib resistance was different between PC9GR and HCC827GR cells. PC9GR cells only acquired the T790M resistance mutation, whereas HCC827GR cells showed *C-MET* amplification (Figure 1B–G). To measure the pre-treatment level of T790M mutation and *C-MET* amplification in parental cancer cell lines, we performed ultra-sensitive ddPCR assays with a sensitivity of 0.01–0.005% that are optimized for detecting minor-frequency gene alterations [17]. No significant difference was found in the pre-treatment level of T790M mutation and *C-MET* amplification between PC9 and HCC827 cells (Figure 1C,E). Regarding the resistance mechanism, PC9GR cells with T790M mutation were sensitive to the third-generation EGFR-TKI AZD9291, while HCC827GR cells with *C-MET* amplification were sensitive to a combination of gefitinib and the MET inhibitor PHA665752 (Figure 1H,I). To confirm this sensitivity to the drug, we checked downstream targets of EGFR and C-MET. In PCR9GR, expressions of phosphorylated AKT and ERK were decreased significantly with downregulated expressions of active EGFR and MET after treatment of AZD9291 (Figure 1H). Moreover, these phosphorylated downstream targets were totally decreased in HCC827GR cells with dual treatment of EGFR and MET-TKIs following the cell death (Figure 1I). Through these results, we concluded that there are different acquired resistance mechanisms according to cell type as shown in PC9GR and HCC827GR.

### 3.2. Resistant Cell Clones Selected by Short-Term Drug Treatment

**Figure 2:**
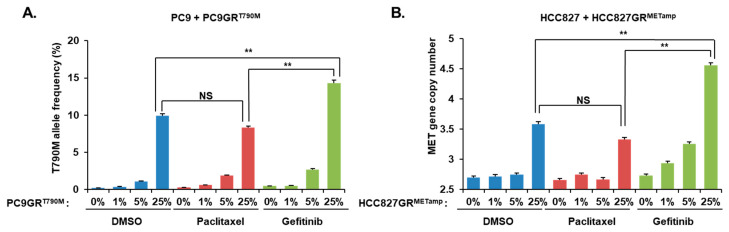
Selection of clones harboring resistant gene alteration in short term EGFR-TKI treatment. EGFR-TKI-resistant cells were spiked into parental EGFR-TKI-sensitive cells at the indicated percentages, and these cell mixtures were treated with DMSO, gefitinib (0.1 µM), for 48 h. (**A**) PC9GR cells were mixed with PC9 cells at the indicated percentages. ddPCR assays were performed to evaluate the allele frequency of *EGFR* T790M mutations. (**B**) HCC827GR cells were mixed with HCC827 cells at the indicated percentages. ddPCR assays were performed to evaluate the *C-MET* gene copy number. In all graphs, the error bars represent the mean ± SEM. Statistical analysis was performed using *t*-test. ** *p* ≤ 0.05; NS, not significant.

We first explored whether molecular-targeted drugs can select cancer cell clones harboring specific resistance gene alterations in cell line mixtures including EGFR-TKI-sensitive and EGFR-TKI-resistant cells. TKI-resistant cells with a T790M mutation (PC9GR) were spiked into TKI-sensitive cells without a T790M mutation (PC9) at various proportions (Figure 2A). The allele frequency of T790M mutation was calculated after 48 h of treatment with DMSO, gefitinib, or paclitaxel in the cell line mixtures. Gefitinib significantly increased the allele frequency of T790M resistance mutations compared with the cytotoxic drug paclitaxel. This finding was repeated in the experiment using cell line mixtures of EGFR-TKI-resistant cells with *C-MET* amplification (HCC827GR) and EGFR-TKI-sensitive cells without *C-MET* amplification (HCC827) (Figure 2B).

**Figure 3:**
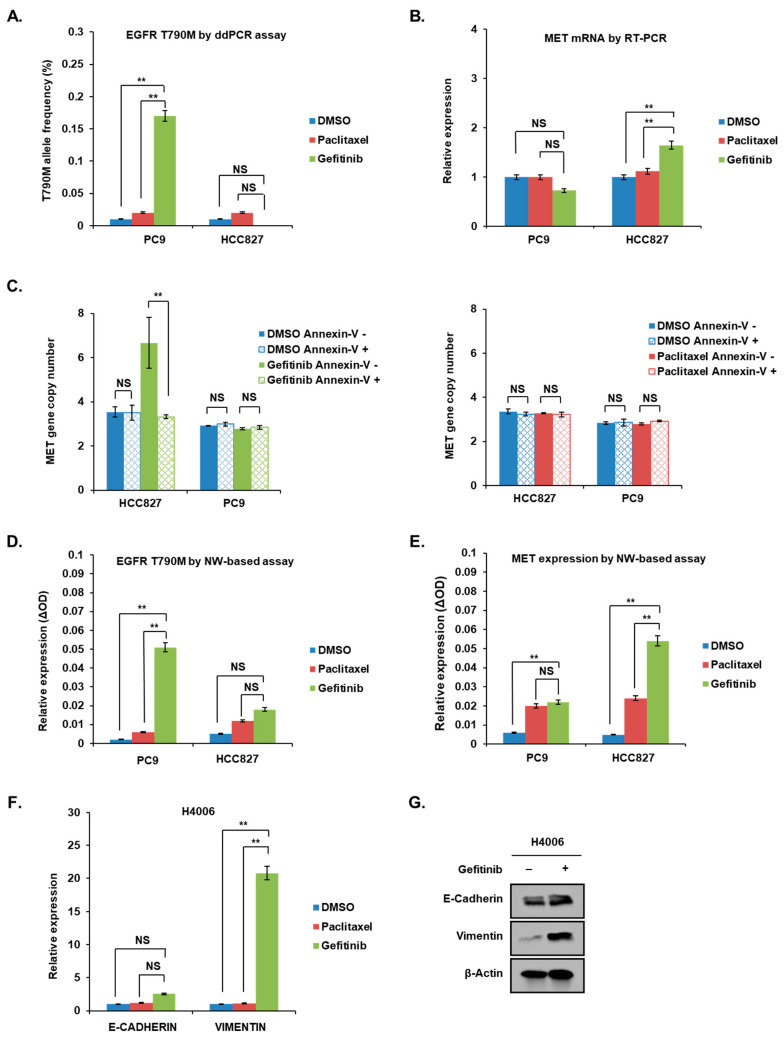
Expression of specific gene alterations related to EGFR-TKI resistant mechanism in different cell lines. In the parental cell lines (PC9 and HCC827), (**A**) *EGFR* T790M mutation levels and (**B**) *C-MET* mRNA expression were measured after 48 h of treatment with DMSO, gefitinib (0.1 µM), or paclitaxel (0.1 µM). The allele frequency of *EGFR* T790M mutation was calculated using the ddPCR assay. *C-MET* mRNA expression was measured by RT-PCR. (**C**) PC9 and HCC827 cells were sorted according to the expression levels of annexin-V-FITC and MET-Alexa 647 after 48 h of treatment with DMSO, gefitinib (0.1 µM), or paclitaxel (0.1 µM). The copy number of *C-MET* gene in each group was measured using the ddPCR assay. (**D**,**E**) The amount of *EGFR* T790M mutation and *C-MET* expression was also measured using nanowire-based (NW) colorimetric assays. (**F**,**G**) In H4006 cells, the mRNA and protein expression levels of E-CADHERIN and VIMENTIN were measured by RT-PCR and Western blotting after 48 h of treatment with gefitinib (0.1 µM). In all graphs, the error bars represent the mean ± SEM. Statistical analysis was performed using *t*-test. ** *p* ≤ 0.05; NS, not significant.

When PC9 cells and HCC827 cells were treated with gefitinib for 48 h, the frequency of T790M mutation increased significantly in PC9 cells but did not change in HCC827 cells (Figure 3A). The antimitotic drug paclitaxel, which efficiently induces apoptosis, did not induce an increased T790M mutation frequency in PC9 cells, consistent with the results of our previous cell line mixture experiments. Notably, T790M mutation was increased only in PC9 cells treated with EGFR-TKI, gefitinib, indicating that T790M mutation is specific for the EGFR inhibitor in these *EGFR*-mutant cells with this pre-existing resistance mutation. However, *C-MET* mRNA expression was significantly increased in HCC827 cells after 48 h of treatment with gefitinib, whereas it was decreased in PC9 cells (Figure 3B). Paclitaxel treatment did not increase *C-MET* mRNA, even in HCC827 cells. We conducted further experiments to confirm whether the increased *C-MET* mRNA expression after short-term gefitinib results from the expansion of pre-existing resistant clones with *C-MET* amplification. The copy numbers of the *C-MET* gene in the Annexin V-negative HCC827 cells that survived shortly after gefitinib treatment were significantly higher than those of the Annexin V-positive dead HCC827cells (Figure 3C). We repeated these short-term treatment experiments using a novel nanowire-based colorimetric genotyping assay. This ctDNA assay isolates highly-fragmented DNA using the nanowire and directly detects DNA variants at the same nanowire platform without PCR amplification; thus, the assay detects very low-abundance DNA variants without false-positive detection [18,19,20,21]. These assays showed that T790M mutations were significantly increased in PC9 cells treated with gefitinib, while *C-MET* gene expression was significantly increased in HCC827 cells treated with gefitinib (Figure 3D–E). Taken together, these results support that pre-existing T790M-positive clones may be selected and expanded following short-term gefitinib exposure of PC9 cells that would develop T790M mutations eventually after long-term EGFR-TKI treatment. In HCC827 cells that acquire *C-MET* amplification as the final gefitinib resistance mechanism, *C-MET*-amplified clones may be expanded even after short-term gefitinib exposure. Thus, the growth of these *C-MET*-related resistant clones may be identified by observing increased *C-MET* expression.

To further validate this finding, we used another EGFR-TKI-sensitive cell line, H4006, which shows EMT without secondary alterations in *EGFR* and *MET* genes when EGFR-TKI resistance occurs [22]. We examined whether the expression of EMT-related genes was altered in H4006 cells after short-term EGFR-TKI treatment. When we treated H4006 cells with gefitinib for 48 h, the mRNA and protein levels of VIMENTIN were significantly increased, whereas those of E-CADHERIN were unchanged (Figure 3F,G). Additionally, neither the T790M mutation frequency nor the *C-MET* mRNA expression was increased (Figure S1).

**Figure 4:**
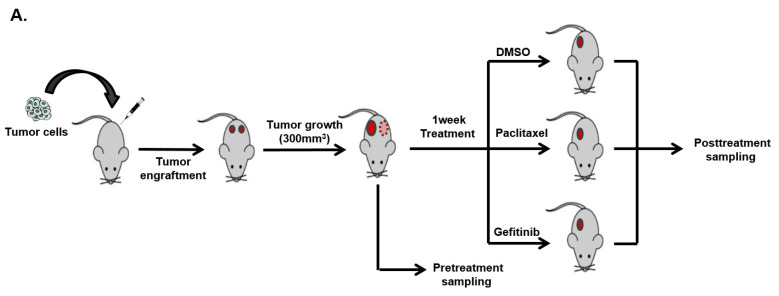

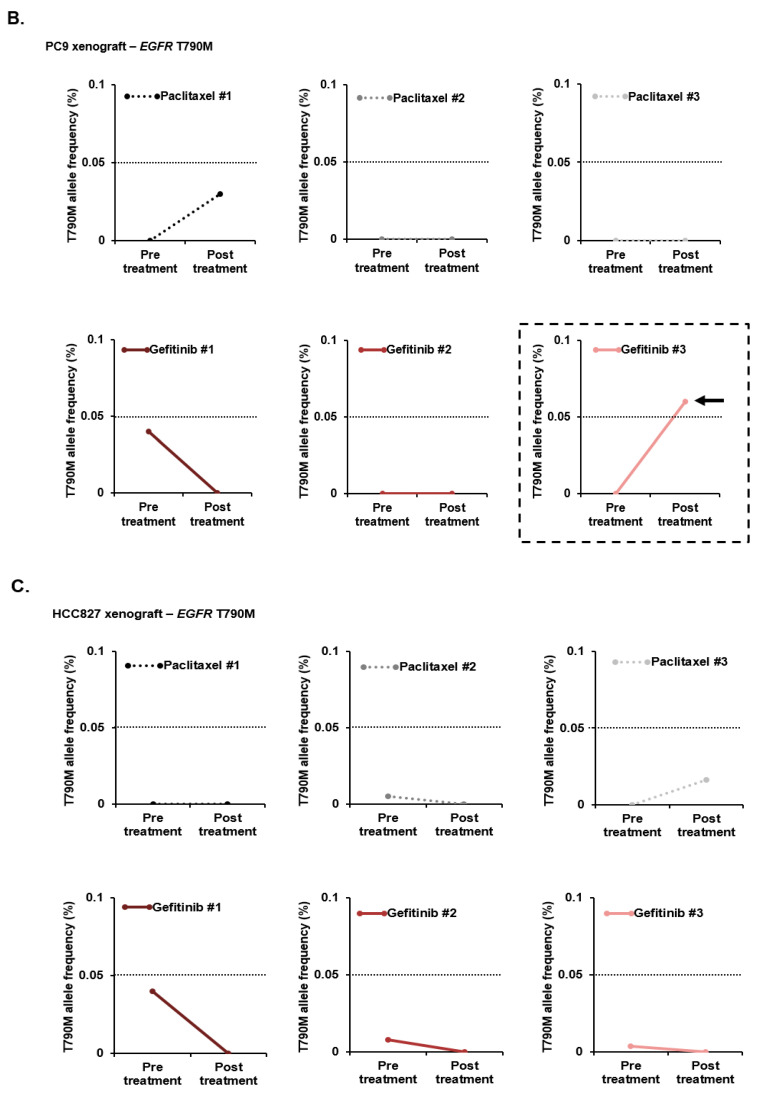

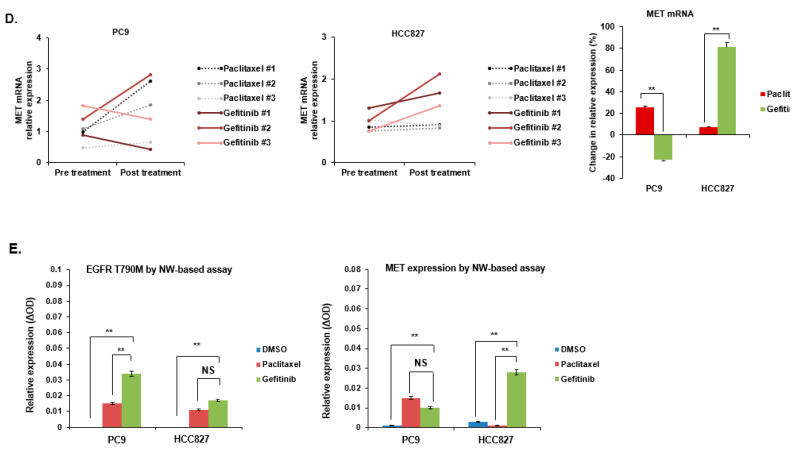
Expression of specific gene alterations related to EGFR-TKI resistant mechanism in vivo. (**A**) Schematic diagram of xenograft experiments. (**B**,**C**) *EGFR* T790M mutation changes in each sub-cutaneous PC9 or HCC827 xenograft tumor after gefitinib or paclitaxel treatment. The T790M mutation allele frequency was measured using the ddPCR assay. The dotted line indicates the threshold for *EGFR* T790M mutation detection (0.05%). (**D**) Change in the *C-MET* mRNA expression level in each subcutaneous PC9 or HCC827 xenograft tumor after gefitinib or paclitaxel treatment. *C-MET* mRNA expression was measured by RT-PCR. (**E**) The levels of *EGFR* T790M mutation and *C-MET* expression were measured using the nanowire-based (NW) colorimetric assay. Error bars represent the mean ± SEM. Statistical analysis was performed using t-test. ** *p* ≤ 0.05; NS, not significant.

To extend these in vitro results, we conducted xenograft studies using cell line models of *EGFR*-mutant lung cancer (HCC827 and PC9). In the xenograft model, the tumor volume, frequency of T790M, and mRNA expression level of *MET* were measured. Consistent with in vitro findings, gefitinib treatment increased T790M mutations above the LOD of that mutation in one of three subcutaneous PC9 xenograft tumors but not in PC9 xenograft tumors treated with paclitaxel or HCC827 xenograft cells treated with paclitaxel or gefitinib (Figure 4A–C). *MET* expression in HCC827 tumors was significantly increased after gefitinib treatment compared with that in the same tumors treated with paclitaxel (Figure 4D). However, *MET* expression was significantly decreased in PC9 xenografts treated with gefitinib. There findings were repeated using the nanowire-based assay (Figure 4E). Based on these findings in different experimental models, the resistance cell clones that increase shortly after initiating targeted treatment vary and are associated with the final overall resistance phenotype.

### 3.3. Prediction of Resistance Using PDCs

**Figure 5:**
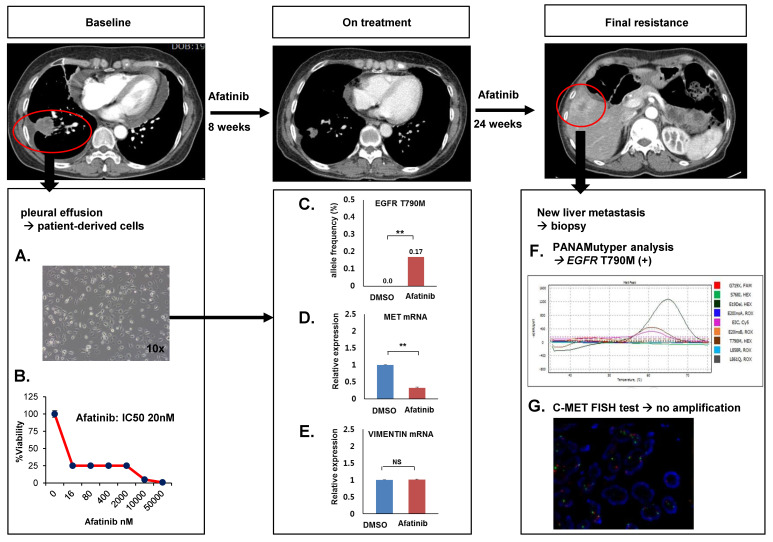
Prediction of resistant gene alteration using PDCs. (**A**) A PDC line (NCCLu-15) was established using pleural effusion from a treatment-naïve patient with lung cancer harboring an EGFR exon 19 deletion. (**B**) Sensitivity to the EGFR-TKI afatinib was tested using the MTS assay. (**C**–**E**) EGFR T790M mutations, C-EMT mRNA expression, and VIMENTIN mRNA expression were measured after 48 h of treatment with DMSO and afatinib (2 µM) in the NCCLu-15. The allele frequency of EGFR T790M mutation was calculated by ddPCR. The C-MET and VIMENTIN mRNA levels were measured by RT-PCR. (**F**,**G**) EGFR T790M mutations and C-MET amplification were evaluated in post-treatment tumor tissue collected after the patient developed resistance to afatinib. EGFR mutations were tested using the PNA-Mediated Real-Time PCR Clamping Method (PANAMutyper), and C-MET amplification was assessed using fluorescence in situ hybridization. Error bars represent the mean ± SEM. Statistical analysis was performed using *t*-test. ** *p* ≤ 0.05; NS, not significant.

To verify whether the early emergence of resistant clones could predict the final resistance mechanism in other lung cancer cell models, we investigated a cell line (NCCLu-15) derived from a treatment-naïve 65-years-old female NSCLC patient with *EGFR* mutation (Figure 5A). This patient was a 58-year-old woman diagnosed with stage IV adenocarcinoma of the lung carrying an *EGFR* exon 19 deletion. She received the EGFR-TKI afatinib as a first-line treatment and showed a partial response. However, her disease progressed after 6 months, and rebiopsy was performed on new liver metastasis. Only an *EGFR* T790M mutation was detected without *C-MET* amplification or EMT in the resistant tumor tissue (Figure 5F,G). We treated initial PDCs (NCCLu-15) with afatinib for 48 h and examined the frequency of T790M mutation and *C-MET* and *VIMENTIN* mRNA expression (Figure 5B–E and Figure S2). In the afatinib-treated NCCLU-15 cells, the frequency of T790M significantly increased, whereas *C-MET* expression decreased and *VIMENTIN* expression was unchanged. These findings revealed that early quantitative resistance-associated gene alterations might predict the final mechanism of resistance to EGFR-TKI in the PDC model.

### 3.4. Prediction of Resistance Using Plasma ctDNA

**Figure 6:**
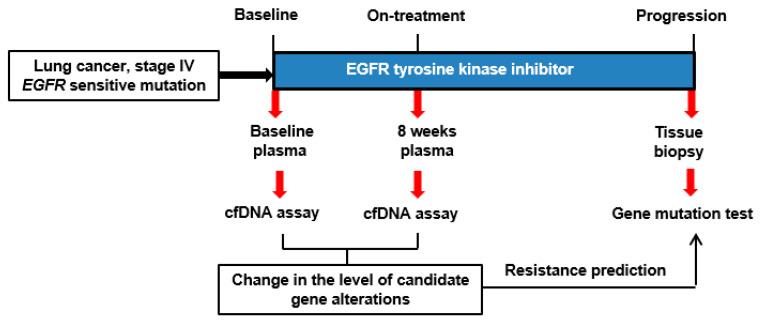
Clinical study flow chart.

A growing body of evidence indicates that ctDNA in the blood can reflect the molecular and genetic features of primary and metastatic tumors [23,24]. Thus, we assessed plasma ctDNA to quantitatively monitor variation in resistance-related clones while *EGFR*-mutated lung cancer patients were treated with EGFR-TKIs. We prospectively collected blood samples from four patients with *EGFR*-mutated NSCLC at baseline and 8 weeks after EGFR-TKI treatment and measured the levels of T790M mutations and *C-MET* expression in plasma ctDNA using the nanowire-based colorimetric assay (Figure 6). In a 46-year-old male patient, the 8-week on-treatment plasma ctDNA level of T790M mutations and *C-MET* expression increased by 89% and 43%, respectively (Table 1). He finally exhibited acquired resistance to gefitinib at 28.5 months, and his resistant tumor showed both T790M mutation and *C-MET* amplification. In a 68-year-old female patient, the 8-week on-treatment plasma ctDNA level of T790M mutations was increased (+167%). Her *C-MET* expression level was slightly increased (+16%) but did not reach the LOD for that gene overexpression. Her final EGFR-TKI resistance mechanism was confirmed as T790M mutation without *C-MET* amplification. In contrast to the above two patients, the other two cases showed no significant on-treatment increase in T790M mutation or *C-MET* expression in their plasma ctDNAs, and neither T790M mutation nor *C-MET* amplification was observed in their post-treatment tissue. No significant difference was found in the baseline ctDNA level of T790M mutations or *C-MET* expression among the four patients.

## 4. Discussion

We first evaluated whether the mechanism of acquired EGFR-TKI resistance varies among individual tumors using two lung cancer cell lines harboring the same *EGFR* mutation. Although two gefitinib-resistant cancer cell lines were established by the same drug and treatment method during the same period, they showed different drug resistance mechanisms. This finding was consistent with that in a study by Shien et al. in which three lung cancer cell lines with *EGFR* mutations became gefitinib-resistant with different mechanisms after stepwise escalation of chronic exposure to gefitinib [25]. In that study, PC9, HCC827, and H4006 cells that acquired gefitinib resistance harbored a secondary T790M mutation, *C-MET* amplification, and EMT features, respectively [25]. However, how the different resistance mechanisms developed during treatment with the same EGFR-TKI was not revealed. One possible explanation is that each tumor with *EGFR* mutations has different innate drug-resistant cell clone profiles. The selection of pre-existing drug-resistant cancer cell clones during treatment is a mechanism of acquired resistance to molecularly targeted drugs [8,10]. A preclinical study by Turke et al. identified *C-MET* amplification as the mechanism of EGFR-TKI resistance and reported subclones of cancer cells carrying *C-MET* amplification before drug exposure [8]. Additionally, one whole-genome sequencing study analyzing several *EGFR*-mutant lung cancers transforming into small cell lung cancer after an initial response to EGFR-TKI demonstrated that small cell lung cancer precursors were already present before treatment [11]. Several clinical studies demonstrated the presence of low-abundance *EGFR* T790M mutations in pre-treatment tumor tissue of patients with NSCLC harboring *EGFR* mutations [13,14,15]. However, the latent drug-resistant cell theory cannot fully explain why different drug resistance mechanisms occur. We found no significant difference in the baseline levels of *EGFR* T790M mutations and *C-MET* gene copy numbers in two parental cancer cells that subsequently developed different gefitinib resistance mechanisms after treatment. Further investigation is needed to evaluate the fundamental causes of different drug resistance mechanisms.

Most pre-existing drug-resistant subclones are present at very low frequencies within a tumor before treatment, and they cannot be detected using standard sequencing methods [8,12]. In addition, multiple resistant clones with different molecular mechanisms may exist within a tumor, and which resistance-associated clones will survive drug treatment cannot be predicted. Thus, we assumed that measuring changes in drug-resistant clone proportions after short-term drug exposure would be more useful to predict the final resistance mechanism than measuring their initial proportion before drug exposure. Consequently, we observed that the patterns in resistance-related genes or gene products after short-term exposure to EGFR-TKI varied depending on *EGFR*-mutant lung cancer cell lines with a different EGFR-TKI resistance mechanism. This finding suggests that early emergent resistance clones can reflect the final molecular mechanism for EGFR-TKI failure.

To validate the preclinical findings, we performed serial plasma ctDNA analysis in four patients during EGFR-TKI treatment. One reason for ctDNA monitoring is that multiple tissue sampling over time is not feasible in most lung cancer patients. In addition, accurately measuring the proportion of gene alterations within a tumor is challenging because the biopsy sample can contain varying numbers of non-tumor cells. Thus, liquid biopsy, such as sampling circulating tumor cells or ctDNA, is more feasible and accurate in tracking molecular changes during anti-cancer treatment. We applied a new ctDNA analysis method, the nanowire-based colorimetric assay, which is an accurate, cost-effective, and rapid method for oncogenic mutations or amplification using small amounts of plasma in patients with several tumor types [18,21]. Increasing levels of *EGFR* T790M or *C-MET* expression in plasma ctDNA after short-term EGFR-TKI treatment was closely related to *EGFR* T790M or *C-MET* amplification in the post-treatment tumor tissue. However, this finding requires further validation studies because of our small sample size. Moreover, further technological improvement is needed to fully use liquid biopsy samples for the early prediction of the EGFR-TKI resistance mechanism. First, multiple specific gene alterations relevant to EGFR-TKI resistance must be simultaneously assessed despite using small biological specimen amounts. Second, very small fractional variations of gene variants should be detected with high accuracy. This approach requires analysis of transcriptomic data as well as genomic data to recognize nongenetic mechanisms related to resistance (e.g., EMT and transformation to small cell lung cancer). Technological progress in both ultra-deep sequencing methods and non-invasive sampling will improve the ability to predict the drug-resistant mechanism during EGFR-TKI treatment. Another promising method for the early prediction of drug-resistant mechanisms in patients who start EGFR-TKI treatment is to use PDC models. PDCs can provide more material to perform comprehensive genetic tests than blood samples. If the time to establish PDC lines can be shortened, their clinical application will be further expanded.

Since EGFR-TKIs were established as a first-line standard of care for *EGFR*-mutant NSCLC patients, several randomized clinical trials in the last decade have evaluated the efficacy of combination treatment with EGFR-TKIs and other drugs [26,27]. In the NEJ009 study, the survival benefit of concurrent treatment with gefitinib and pemetrexed-carboplatin chemotherapy with gefitinib alone as a first-line treatment was compared in Japanese patients with advanced NSCLC with *EGFR* mutations [26]. An unprecedented median overall survival (OS) of 52.2 months after EGFR-TKI gefitinib and chemotherapy combination was reported, significantly longer than the 38.8-month median OS after treatment with gefitinib alone. In addition, a combination of erlotinib plus the anti-angiogenesis agent bevacizumab showed excellent outcomes compared with erlotinib alone (median progression-free survival (PFS), 16.9 vs. 13.3 months; hazard ratio (HR) = 0.605; *p =* 0.016) [27]. This combination strategy demonstrated survival outcomes comparable to the outstanding results of the third-generation EGFR-TKI osimertinib as a first-line standard treatment in the FLAURA study (median PFS, 18.9 vs. 10.2 months, HR = 0.46, *p* < 0.001; median OS, 38.6 vs. 31.8 months, HR = 0.80, *p* = 0.046) [28]. However, these combination treatment strategies do not reflect individual biological characteristics of lung cancer with *EGFR* mutations. A combination treatment that simultaneously targets *EGFR* mutation and emerging resistance mechanisms is a reasonable personalized strategy to further improve patient outcomes. The early prediction of resistance mechanisms would be essential for the success of this treatment strategy.

## 5. Conclusions

In conclusion, acquired drug resistance is a crucial practical challenge during EGFR-TKI treatment of advanced NSCLC patients with *EGFR* mutations. Our study revealed that early quantitative changes of resistance-related genes or gene products might reflect the final mechanism of EGFR-TKI resistance in tumors carrying *EGFR* mutations. Specifically, the early proportional variation of resistance gene mutations in plasma ctDNA was closely associated with the final resistance mechanism observed in tumor tissues. Predicting drug resistance early during treatment can facilitate the development of personalized therapy, including adding other therapeutic agents, blocking bypass pathways, or changing to more effective EGFR-blocking agents in individual patients harboring *EGFR* mutations. Adopting this treatment strategy for *EGFR*-mutant lung cancer may prevent or delay EGFR inhibitor resistance and improve patient survival outcomes.

## Figures and Tables

**Table 1:** The association between the on-treatment plasma cell-free DNA level of gene alterations and final resistance mechanism for EGFR-targeted inhibitors.

	Age	Sex	Founder EGFR Mutation	Drug	PFS (Month)	On-Treatment Plasma Cell-Free DNA Assay *						Posttreatment Tissue Test	
						EGFR T790M Mutation			C-MET Expression			EGFR T790M Mutation ^†^	MET Amplification ^§^
						Baseline (ΔOD)	8 Weeks (ΔOD)	Change	Baseline (ΔOD)	8 Weeks (ΔOD)	Change		
1	46	M	19del	gefitinib	28.5	0.018	0.034	+89%	0.021	0.030	+43%	Positive	Positive
2	68	F	19del	gefitinib	57.3	0.009	0.024	+167%	0.010	0.012	+16%	Positive	Negative
3	62	F	19del	gefitinib	20.8	0.007	0.006	−14%	0.010	0.009	−10%	Negative	Negative
4	58	M	19del	gefitinib	17.8	0.014	0.009	−36%	0.006	0.007	−17%	Negative	Negative

Note: * Nanowire-based plasma cell-free DNA assay, ^†^ PNA Mediated Real-time PCR Clamping Method, and ^§^ Fluorescence in situ hybridization test. Abbreviation: PFS, progression-free survival.

## Data Availability

The data sets used and/or analyzed during the current study are available from the corresponding author on reasonable request.

## Supplementary Materials

The following supporting information can be downloaded at: www.mdpi.com/article/10.3390/cancers14061512/s1, Figure S1: Expression of specific gene alterations related to EGFR-TKI resistant mechanism in H4006; Figure S2: Expression of *EGFR* T790M mutation in PDCs; Table S1: Primer sequences for direct sequencing of *EGFR* T790M; Table S2: Primer sequences for qRT-PCR; Table S3: Biotinylated probe sequences.
