# Supplementary material for: Early On-Treatment Prediction of the Mechanisms of Acquired Resistance to EGFR Tyrosine Kinase Inhibitors
Source: Cancers (Basel). 2022 Mar 15;14(6):1512. doi: 10.3390/cancers14061512 (PMC8946020; doi:10.3390/cancers14061512)
Supplement: Supplementary file 1 [file cancers-14-01512-s001.zip › Supplementary Figures and Tables (with caption).pdf]

A.

Sample		T790M	Conc.	+	-	A.D	F.A
H4006	DMSO	HEX	253	2867	11962	14829	
		FAM	0	0	14829		
	Gefitinib	HEX	257	3060	12546	15606	
		FAM	0	0	15606		
Control							
NTC		FAM	0	0	13733	13733	
		HEX	0	0	13733		
A549		HEX	12.8	172	15705	15877	
		FAM	0	0	15877		
H1975		HEX	14	201	16763	16964	72.7
		FAM	37.3	530	16434		

B.

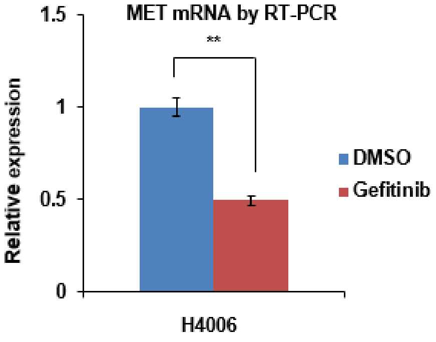

Figure S1. Expression of specific gene alterations related to EGFR-TKI resistant mechanism in H4006. (**A**) *EGFR* T790M mutation changes in H4006 after gefitinib treatment. The T790M mutation allele frequency was measured using the ddPCR assay. NTC: Negative Template Contorl; A549: T790M negative cell; H1975: T790M positive cell. (**B**) Change in the *C-MET* mRNA expression level in H4006 after gefitinib treatment. *C-MET* mRNA expression was measured by RT-PCR. Error bars represent the mean ± SEM. Statistical analysis was performed using t-test. ** $p \leq 0.05$; ns, not significant.

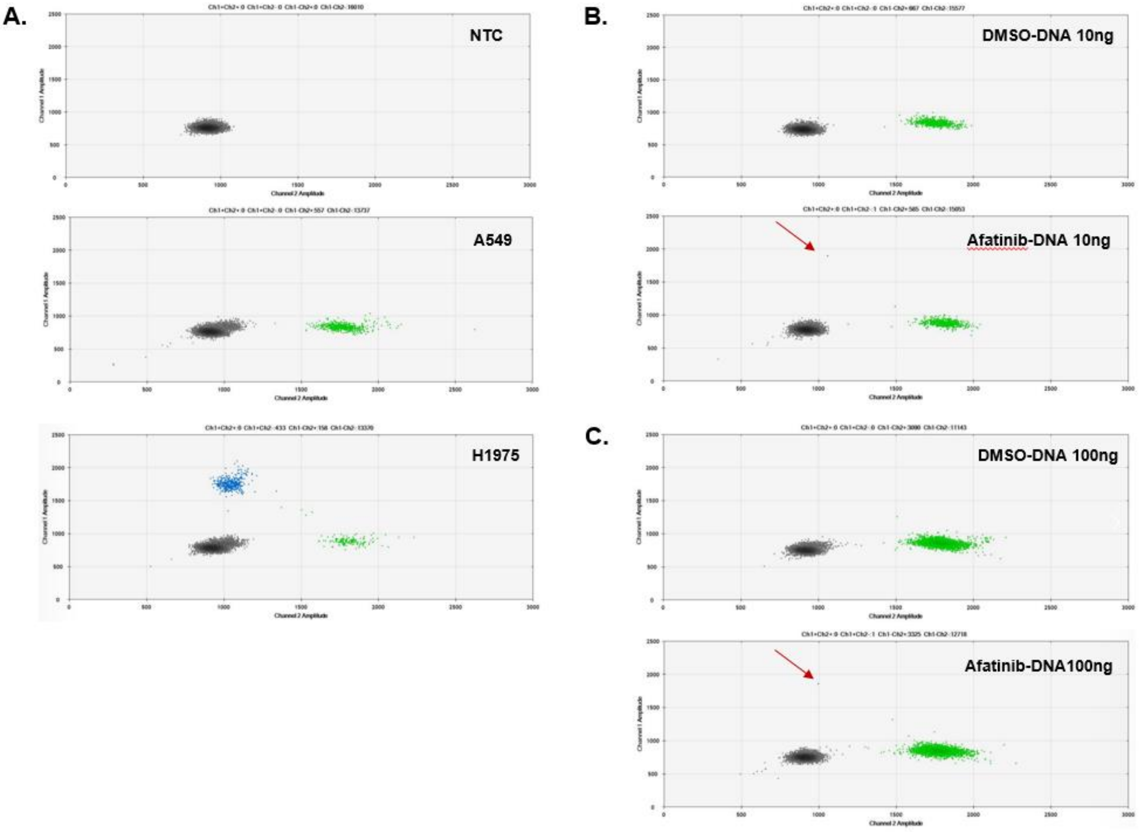

Figure S2. Expression of *EGFR* T790M mutation in PDCs. (**A**) *EGFR* T790M mutation changes in experimental controls. NTC: Negative Template Contorl; A549: T790M negative cell; H1975: T790M positive cell. (**B**) *EGFR* T790M mutation changes in PDCs after afatinib treatment. The T790M mutation allele frequency was measured using the ddPCR assay. Red arrow indicate that *EGFR* T790M positive oil-droplet.

Table S1. Primer sequences for direct sequencing of *EGFR* T790M

EGFR T790M	F-5'-CCATGAGTACGTATTTTGAAACTC-3'<br>R-5'-CATATCCCCATGGCAAACTCTTGC-3'

Table S2. Primer sequences for qRT-PCR

MET	F-5’-TGCACAGTTGGTCCTGCCATGA-3’<br>R-5’-CAGCCATAGGACCGTATTTCGG-3’
VIMENTIN	F-5’-AGGCAAAGCAGGAGTCCACTGA-3’<br>R-5’-ATCTGGCGTTCCAGGGACTCAT-3’
E-CADHERIN	F-5’-GCCTCCTGAAAAGAGAGTGGAAG-3’<br>R-5’-TGGCAGTGTCTCTCCAAATCCG-3’
GAPDH	F-5’-TGATGACATCAAGAAGGTGG-3’<br>R-5’-TCCTTGGAGGCCATGTGGGC-3’

Table S3. Biotinylated probe sequences

Probe label	Sequence
T790M probe 1	5′ – biotin – CCA CCG TGC AGC TCA TCA TGC A – 3′
T790M probe 2	5′ – GCA AGA GTT TGC CAT GGG GAT ATG – biotin 3′
C-MET probe 1	5’ – Biotin-TGCACCCCTTGAAGGAGGGACAAG – 3’
C-MET probe 2	5’ – CGAAATCCAAAGTCCCAGCCAC-Biotin – 3’
C-MET probe 3	5‘– Biotin-CAGTCAAGGTTGCTGATTTTGGT – 3’
C-MET probe 4	5’ – ATAGTATTCTTTATCATACATGT-Biotin – 3’
